# Adherence to the Mediterranean diet moderates the association of aminotransferases with the prevalence of the metabolic syndrome; the ATTICA study

**DOI:** 10.1186/1743-7075-6-30

**Published:** 2009-07-30

**Authors:** Natalia Tzima, Christos Pitsavos, Demosthenes B Panagiotakos, Christina Chrysohoou, Evangelos Polychronopoulos, John Skoumas, Christodoulos Stefanadis

**Affiliations:** 1 Department of Nutrition Science-Dietetics, Harokopio University, Athens, Greece; 2 First Cardiology Clinic, School of Medicine, University of Athens, Athens, Greece

## Abstract

**Background:**

Elevated liver enzymes are markers of liver steatosis and metabolic syndrome. We aimed to investigate the association of Mediterranean diet on the relationship between aminotransferases (i.e., AST, ALT, gGT) and the metabolic syndrome.

**Methods:**

The ATTICA study has randomly enrolled 1514 adult males (18–87 yrs) and 1528 females (18–89 yrs) from the greater area of Athens. Adherence to Mediterranean diet was assessed through the MedDietScore. According to NCEP III criteria, participants were classified into those with or without the metabolic syndrome.

**Results:**

Women with metabolic syndrome had higher γGT (p = 0.02) and lower AST/ALT levels (p = 0.018) than those without, and men with metabolic had a lower AST/ALT ratio (p = 0.01) compared to those without metabolic syndrome. The AST/ALT ratio was also positively correlated with MedDietScore (rho = 0.17, *p *< 0.001), while higher MedDietScore was associated with lower likelihood of having the metabolic syndrome in a multi-adjusted analysis (OR = 0.34, 95% CI: 0.16–0.73). Stratified analysis by the level of adherence to the Mediterranean diet, revealed that only in subjects away or with moderate adherence to the Mediterranean diet, an increase in the AST/ALT ratio was associated with lower likelihood of having the metabolic syndrome (OR = 0.33, *p *< 0.05 and OR = 0.34, *p *< 0.09, respectively); however, when we focused in those with greater adherence to the Mediterranean diet, AST/ALT ratio was not associated with the presence of the syndrome (OR = 0.51, *p *= 0.55). These findings remained similar in both genders, and even when the quantity of alcohol drinking was taken into account.

**Conclusion:**

Aminotransferases ratio constitutes a marker of the metabolic syndrome among healthy adults; however, this relationship is moderated when individuals are close to the Mediterranean dietary pattern.

## Introduction

The metabolic syndrome is a combination of metabolic disorders, such as dyslipidemia, hypertension, impaired glucose tolerance, compensatory hyperinsulinemia and the tendency to develop fat around the abdomen [1]. People with metabolic syndrome are at high risk for atherosclerosis and, consequently, cardiovascular disease.

Alanine aminotranferase (ALT), aspartate aminotransferase (AST) and gamma -glutamyltransferase (γGT) are common markers of liver injury. Non alcoholic fatty liver disease (NAFLD) constitutes the most frequent explanation for abnormal liver tests results and accounts for asymptomatic elevation of aminotransferase levels in up to 90% of cases. [2,3] It is proposed that NAFLD may be part of a wider coexistence of features, besides the core set of disorders which characterize the metabolic syndrome and liver enzymes-NAFLD associated biomarkers have indeed been correlated with metabolic syndrome, its components and cardiovascular disease. [4-8] Among the different liver enzymes, ALT has been related with hepatic fat deposition and insulin resistance which plays a major role in metabolic syndrome. [9] In cases where AST/ALT ratio has been used instead of ALT, it has been found that this ratio is also correlated with metabolic syndrome and insulin resistance [10,11] AST/ALT ratio could be useful for distinguishing the etiology and severity of different liver damage as a value >2 may indicate advanced alcohol liver disease and on the other hand, AST/ALT <1 may imply fat liver accumulation and NAFLD. [12-14] Nevertheless, the exact role of AST/ALT ratio in assessing metabolic syndrome is unclear.

Previous data from the ATTICA and other studies have shown that Mediterranean diet is related with lower prevalence of the metabolic syndrome. [15,16] However, the underlying mechanisms are not fully understood. Ôhe effect of this type of diet on liver enzymes and NAFLD is barely explored, so it is not yet investigated such a possible mediator for the effect of Mediterranean diet to the presence of the metabolic syndrome. [17] Therefore, the goal of the present work was to evaluate whether serum circulating liver markers, like γ-GT, AST, ALT and AST/ALT ratio are associated with the presence of the metabolic syndrome, in a Greek adult population, free from cardiovascular disease. Furthermore, we sought to investigate whether adherence to the Mediterranean diet plays a role in the relationship of circulating liver markers with metabolic syndrome.

## Methods

### Participants of the study

The ATTICA study is a health and nutrition survey, which is being carried out in the province of Attica (including 78% urban and 22% rural areas), where Athens, is a major metropolis. [18] The sampling was random, multistage and based on the age-sex distribution of the province of Attica provided by the National Statistical Service (census of 2001). The sampling anticipated enrolling only people without any clinical history of cardiovascular disease or any other atherosclerotic disease. People who reported chronic viral hepatitis or other known cause of chronic liver disease were excluded from the study. Participants did not have cold or flu, acute respiratory infection, dental problems or any type of surgery during the past weeks. From May 2001 to December 2002, 4056 inhabitants from the above area were randomly selected and of them, 3042 agreed to participate (75% participation rate); 1514 males (18–87 years) and 1528 females (18–89 years). The selected sample can be considered as representative since there were only minor, insignificant, differences in sex and age distribution between the study- and the target population.

All participants were interviewed by trained personnel who used a standard questionnaire that evaluated lifestyle habits and various socio-demographic, clinical and biological characteristics. The study was approved by the Ethics Committee of our Institution and was carried in accordance with the declaration of Helsinki (1989) of the World Medical Association.

### Dietary assessment

Consumption of non-refined cereals and products, vegetables, legumes, fruits, olive oil, dairy products, fish, pulses, nuts, potatoes, eggs, sweets, poultry, red meat and meat products were measured as an average per week during the past year through a validated food-frequency questionnaire (the EPIC-Greek) [19]. The frequency of consumption was then quantified approximately in terms of the number of times a month a food was consumed. Alcohol consumption was measured by daily ethanol intake, in wineglasses (100 ml and 12% ethanol concentration). The Mediterranean dietary pattern [20] consists of: (a) daily consumption: of non refined cereals and products (whole grain bread, pasta, brown rice, etc), vegetables (2 – 3 servings/day), fruits (6 servings/day), olive oil (as the main added lipid) and dairy products (1 – 2 servings/day), (b) weekly consumption: of fish (4–5 servings/week), poultry (3 – 4 servings/week), olives, pulses, and nuts (3 servings/week), potatoes, eggs and sweets (3 – 4 servings/week) and monthly consumption: of red meat and meat products (4 – 5 servings/month). It is, also, characterized by moderate consumption of wine (1 – 2 wineglasses/day) and high monounsaturated: saturated fat ratio (> 2). To evaluate the level of adherence to the Mediterranean dietary pattern a special diet score was used, the MedDietScore [21]; the score has been found reliable, repeatable and valid, in previous works [22]. In particular, for the consumption of food items that are close to this dietary pattern we assigned score 0 for rare or no consumption, 1 for 1 to 4 times/month, 2 for 5 to 8 times, 3 for 9 to 12 times/month, 4 for 13 to 18 times/month and 5 for almost daily consumption. On the other hand, for the consumption of foods that are away from this traditional diet, like meat and meat products, we assigned the opposite scores (i.e. 0 for almost daily consumption to 5 for rare or no consumption). For alcohol, we assigned score 5 for consumption of less than 3 wineglasses/day and, progressively, score 0 for consumption of more than 7 wineglasses/day. Thus, the range of the diet score is between 0 – 55. Higher values of the suggested dietary score indicate greater adherence to the traditional Mediterranean diet.

### Biochemical measurements

The blood samples were collected from the antecubital vein between 8 to 10 a.m., in a sitting position after 12 hours of fasting and avoiding of alcohol. Serum was harvested immediately after admission. The biochemical evaluation was carried out in the same laboratory that followed the criteria of the World Health Organization Lipid Reference Laboratories. ALT, AST and γ-GT but also blood lipids were measured by using chromatographic enzymic method in an automatic analyzer (RA-1000, Dade Behring, Marburg, Germany). The intra-assay and inter-assay coefficient of variation was <5% for ALT, AST and γ-GT. An internal quality control was in place for assessing the validity of cholesterol, triglyceride and HDL methods. The intra and inter-assay coefficients of variation of cholesterol levels did not exceed 9%, triglycerides 4% and HDL 4%. Blood glucose levels were measured with a Beckman Glucose Analyzer. The intra-assay coefficient of variation was 9% and the limit of detection was 3 μU/mL.

### Demographic, clinical and lifestyle characteristics

The study's questionnaire also included demographic characteristics like age, gender, and education level in years of school. Information about smoking habits was collected using a standardized questionnaire developed for the Study. Current smokers were defined as those who smoked at least 1 cigarette per day, former smokers were defined as those who had stopped smoking more than 1 year previously and the rest were defined as non-current smokers. For the ascertainment of physical activity status we used the International Physical Activity Questionnaire (IPAQ), as an index of weekly energy expenditure using frequency (times per week), duration (in minutes per time) and intensity of sports or other habits related physical activity (in MET/minutes) [23]. Participants who did not report any physical activities were defined as sedentary.

Body mass index (BMI) was calculated as weight (in kilograms) divided by height (in meters) squared. Based on the World Health Organization classification, overweight was defined as BMI between 25 and 29.9 kg/m^2^, and obesity was defined as BMI 30 kg/m^2^. [24] We also measured waist circumference (in centimetres) in the middle between the 12^th ^rib and iliac crest. Arterial blood pressure was measured three times at the end of the physical examination with subject in sitting position. Participants whose average blood pressure levels were greater or equal to 140/90 mmHg or were under antihypertensive medication were classified as hypertensive subjects [25]. Hypercholesterolemia was defined as total serum cholesterol levels greater than 200 mg/dL or the use of lipid-lowering agents and diabetes mellitus as a blood sugar > 125 mg/dL or the use of antidiabetic medication [26].

### Definition of the metabolic syndrome

Participants were classified as having the metabolic syndrome or not, according to the definition provided by the National Cholesterol Education Panel -NCEP ATP III, consequently, if 3 or more of the following metabolic components are present: waist circumference ≥ 102 cm for males or ≥ 88 cm for females; triglyceride level ≥ 150 mg/dl; HDL cholesterol level <40 mg/dL for males or <50 mg/dL for females; blood pressure ≥ 130/85 mmHg; fasting blood glucose ≥ 100 mg/dL. [27]

### Statistical analysis

Continuous variables are presented as mean values ± standard deviation, while categorical variables are presented as absolute number and frequencies. Associations between categorical variables were tested by the use of contingency tables and the calculation of chi-squared test. Differences between groups of study in normally distributed continuous variables were evaluated by the calculation of Student's t-test, while the Mann-Whitney test was used for the skewed variables. Correlations between biochemical markers and other continuous variables were tested using the Spearman or the Pearson's correlation coefficients. Binary and ordinal logistic regression analysis was used to evaluate the effect of selected aminotranferases levels on the likelihood of having the metabolic syndrome, as well as the number of metabolic disorders. The Wald statistic was used to hierarchy the effect of aminotranferases on the number of metabolic disorders in the ordinal logistic regression analyses. Multi-adjusted analysis has taken into consideration variables as age, sex, BMI, smoking habits, physical activity status and MedDietScore. Hosmer-Lemeshow statistic was used to evaluate models; goodness-of-fit. All reported *p*-values are based on two-sided tests and compared to a significance level of 5%. SPSS 14.0 software (SPSS Inc. 2002, Chicago, Illinois, USA) was used for all the statistical calculations.

## Results

### Participants' characteristics, liver enzymes and the metabolic syndrome

AST levels were higher in males compared to females (27 ± 12 vs. 24 ± 11 IU/L, p = 0.001); similarly ALT levels were higher in males (24 ± 14 vs. 18 ± 10 IU/L, p = 0.001), as well as γ-GT levels (males: 27 ± 14 vs. females: 18 ± 13 IU/L, p = 0.001). The AST/ALT ratio was lower in males (1.2 ± 0.4 vs. 1.4 ± 0.6, p = 0.02). Moreover, ALT, AST and γ-GT levels were positively correlated with BMI (r = 0.14, p = 0.001, and r = 0.29, p = 0.001 and respectively r = 0.35, p = 0.001), while no associations were found regarding age, smoking habits and physical activity status of the participants.

Table 1 illustrates various characteristics of the participants according to the metabolic syndrome status. Both males and females with metabolic syndrome were more likely to be older, with higher BMI, lower MedDietScore and a greater prevalence of hypertension, hypercholesterolemia and diabetes. In males, γ-GT, AST and ALT concentrations did not differ statistically between the two groups and only a lower AST/ALT ratio was found in males with metabolic syndrome. In females, γ-GT was higher and AST/ALT ratio lower in those with metabolic syndrome, AST levels were similar between the two groups and ALT did not differ statistically.

**Table 1:** Demographic, lifestyle, and clinical characteristics of males and females with and without the metabolic syndrome

Males, *n = 1514*	Metabolic syndrome	No metabolic syndrome	*p*
*Subjects, n*	*379*	*1135*	

Age, years	50 ± 11	44 ± 13	< 0.001
Physical inactivity *(n)*, %	*(136) *36	*(488)*43	0.002
Current smoking *(n)*, %	*(174) *46	*(533) *47	0.81
Med Diet score (0–55)	23.4 ± 6.1	24.5 ± 5.5	0.01
Hypertension *(n)*, %	*(242) *64	*(306) *27	< 0.001
Hypercholesterolemia *(n)*, %	*(216) *57	*(420) *37	< 0.001
Diabetes *(n)*, %	*(72) *19	*(45) *4	< 0.001
Overweight *(n)*, %	*(208) *56	*(533) *47	< 0.001
Obesity *(n)*, %	*(167) *44	*(136) *12	< 0.001
Body mass index (kg/m^2^)	29.8 ± 0.2	26.5 ± 0.1	< 0.001
γ-GT (IU/L)	28 ± 12	26 ± 14	0.24
AST (IU/L)	27 ± 14	27 ± 11	0.98
ALT (IU/L)	26 ± 17	23 ± 12	0.25
AST/ALT ratio	1.11 ± 0.3	1,28 ± 0,48	0.01

Females, *n = 1528*	Metabolic syndrome	No metabolic syndrome	*p*

*Subjects, n*	*228*	*1300*	

Age, years	53 ± 14	43 ± 14	< 0.001
Physical inactivity *(n)*, %	*(62) *27	*(520) *40	< 0.001
Current smoking *(n)*, %	*(78) *34	*(520) *40	0.07
Med Diet score (0–55)	24 ± 6.8	28.2 ± 7.2	< 0.001
Hypertension *(n)*, %	*(128) *56	*(234) *18	< 0.001
Hypercholesterolemia *(n)*, %	*(114) *50	*(442) *34	< 0.001
Diabetes *(n)*, %	*(52) *23	*(39) *3	< 0.001
Overweight *(n)*, %	*(87) *38	*(377) *29	< 0.001
Obesity *(n)*, %	*(105) *46	*(130) *10	< 0.001
Body mass index (kg/m^2^)	29.8 ± 4.9	24.5 ± 4.3	< 0.001
γ-GT (IU/L)	21 ± 12	17 ± 13	0.02
AST (IU/L)	23 ± 9	23 ± 11	0.76
ALT (IU/L)	20 ± 12	18 ± 10	0.18
AST/ALT ratio	1.26 ± 0.3	1.44 ± 0.6	0.018

Continuous variables are presented as mean values ± standard deviation, while categorical variables are presented as absolute number and frequencies.

Unadjusted analysis revealed that 1 unit increase in AST/ALT ratio was associated with 0.37-times (95%CI 0.16–0.84) lower likelihood of having the syndrome in males and with 0.48-times lower likelihood in females (95%CI 0.28–0.86). Greater adherence to the Mediterranean diet was associated with lower likelihood of having the metabolic syndrome (OR per 1 unit increase in diet score in males = 0.97, 95%CI 0.95–0.99, and OR per 1 unit increase in females = 0.90, 95%CI 0.88–0.92). Furthermore, the number of metabolic components of the participants was positively correlated with γ-GT (rho = 0.39, p < 0.001), ALT (rho = 0.22, p < 0.001) concentrations and inversely correlated with AST/ALT ratio (rho = -0.20, p < 0.001). Further trend analysis, after adjusting for age, sex, physical activity and smoking habits revealed that γ-GT (Wald test = 16.79, p < 0.001), followed by ALT (Wald test = 16.27, p < 0.001), AST/ALT ratio (Wald test = 11.82, p = 0.001), were positively associated with the number of metabolic components of the studied sample, while AST (Wald test = 2.41, p = 0.12) showed no significant effect. No gender interactions were observed.

A multi-adjusted analysis, taking into consideration age, sex, BMI, smoking habits, physical activity status and MedDietScore, revealed that a higher AST/ALT ratio was associated with lower likelihood of having the metabolic syndrome (OR = 0.34, 95%CI 0.16–0.73). Additionally, when AST/ALT ratio was categorized in quartiles (males: < 0.93, 0.93–1.16, 1.16–1.43 and >1.43 and females: <1.06, 1.06–1.31, 1.31–1.64 and >1.64), only participants belonging in the highest quartile of AST/ALT ratio were less likely to have the metabolic syndrome, compared to those in the lowest quartile (males: OR = 0.22, 95%CI 0.06–0.80 and females: OR = 0.34, 95%CI 0.13–0.91). No significant associations were observed when AST, ALT markers were tested separately for the presence of the syndrome (*p *= 0.29 and *p *= 0.30, respectively). Furthermore, no significant interaction effect was observed between aminotransferases levels and gender, on the investigated outcome (p = 0.87). Thus, it was decided to perform a pooled analysis with genders combined, in order to increase the statistical power of the analyses.

### Liver enzymes and Mediterranean diet score

Table 2 demonstrates the associations of liver enzymes and other characteristics of the participants with the MedDietScore categories. ALT, AST and γ-GT were inversely associated with the level of adherence to the Mediterranean diet in females, but not in males.

**Table 2:** Demographic, lifestyle, and clinical characteristics of males and females by tertile of Mediterranean diet score

	Mediterranean Diet score			
Males, *n = 1514*	Low	Moderate	High	*p*
*Subjects, n*	767	587	160	

Age, years	53 ± 12	40 ± 8	29 ± 9	< 0.001
Physical inactivity *(n)*, %	*(314) *41	*(235) *40	*(80) *50	0.059
Current smoking *(n)*, %	*(330)*43	*(299)*51	*(80)*50	0.005
Metabolic syndrome *(n)*, %	36	16	8	<0.001
Hypertension *(n)*, %	*(368)*48	*(164) *28	*(19)*12	< 0.001
Hypercholesterolemia *(n)*, %	*(360)*47	*(246)*42	*(29)*18	< 0.001
Diabetes *(n)*, %	*(100)*13	*(17)*3	*(2)*1	< 0.001
Overweight *(n)*, %	*(409) *53	*(358) *61	*(27) *24	< 0.001
Obesity *(n)*, %	*(272) *36	*(28) *5	*(1) *1	< 0.001
Body mass index (kg/m^2^)	29 ± 4	26 ± 2	23 ± 2	< 0.001
γ – GT (IU/L)	27 ± 14	27 ± 9	19 ± 7	0.059
AST (IU/L)	27 ± 13	27 ± 9	25 ± 12	0.74
ALT (IU/L)	24 ± 14	25 ± 12	21 ± 13	0.45
AST/ALT ratio	1.28 ± 0.45	1.17 ± 0.43	1.33 ± 0.46	0.19

Females	Low	Moderate	High	*p*
*Subjects, n*	246	428	854	

Age, years	60 ± 13	53 ± 10	36 ± 10	< 0.001
Physical inactivity *(n)*, %	*(79)*32	*(150)*35	*(359)*42	0.08
Current smoking *(n)*, %	*(61)*25	*(163)*38	*(376)*44	< 0.001
Metabolic syndrome *(n)*, %	38	24	4	<0.001
Hypertension, *(n)*, %	*(125)*51	*(154)*36	*(77)*9	< 0.001
Hypercholesterolemia *(n)*,%	*(130)*53	*(210) *49	*(25)*3	< 0.001
Diabetes *(n)*, %	*(44)*18	*(38)*9	*(8) *1	< 0.001
Overweight *(n)*, %	*(84)*34	*(247) *58	*(131) *15	< 0.001
Obesity *(n)*, %	*(139) *57	*(89) *21	*(8) *1	< 0.001
Body mass index (kg/m^2^)	31 ± 5	27 ± 3	22 ± 2	< 0.001
γ – GT (IU/L)	20 ± 10	20 ± 18	16 ± 8	0.005
AST (IU/L)	24 ± 9	25 ± 13	22 ± 9	0.01
ALT (IU/L)	19 ± 8	20 ± 14	16 ± 8	0.001
AST/ALT ratio	1.31 ± 0.45	1.39 ± 0.71	1.44 ± 0.53	0.29

Continuous variables are presented as mean values ± standard deviation, while categorical variables are presented as absolute number and frequencies.

Although unadjusted aminotransferase ratio did not differ between lower and higher tertile of MedDietScore, further data analysis in the whole population, after adjustment for BMI and alcohol drinking, revealed a positive correlation with AST/ALT ratio (rho = 0.17, *p *< 0.001) and an inverse correlation with AST (rho = -0.11, *p *= 0.03) and ALT levels (rho = -0.22, *p *< 0.001). Moreover, a modified version of the MedDietScore without alcohol intake was also positively correlated with AST/ALT ratio (rho = 0.17, *p *< 0.001), while it was inversely correlated with AST (rho = -0.11, *p *= 0.03) and ALT levels (rho = -0.22, *p *< 0.001). The previous findings suggest that greater adherence to the Mediterranean dietary prototype is associated with reduced aminotranferases levels, and lower ALT concentrations relatively to AST.

### Liver enzymes, Mediterranean diet and the metabolic syndrome

**Figure 1:**
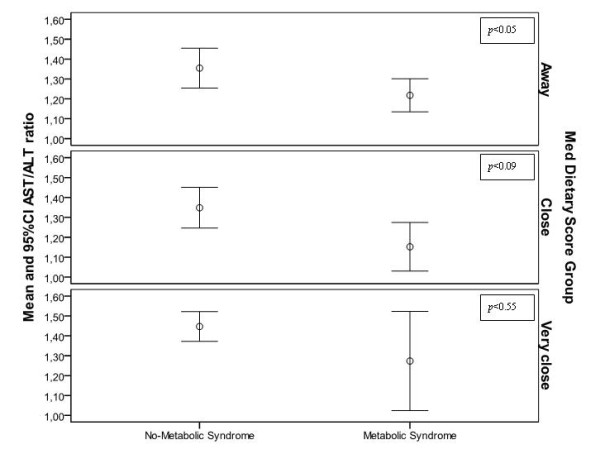
**AST/ALT ratio and the metabolic syndrome, by the level of adherence to the Mediterranean diet**.

However, a highly significant interaction was observed between AST/ALT ratio and MedDietScore (*p *< 0.001). In Figure 1, the crude effect of Mediterranean diet in the association of AST/ALT ratio with metabolic syndrome is illustrated.

Thus, we stratified our analysis by tertile of the diet score (i.e., lower tertile characterizes people away from this traditional dietary pattern, middle and highest tertiles which describe people with moderate and good adherence to the Mediterranean diet). In Table 3 the results from the logistic regression models that evaluated the association between liver enzymes and presence of metabolic syndrome, by the level of adherence to the Mediterranean diet, are presented. Multi-adjusted analysis showed that among people in the lowest and middle tertiles of the diet score, higher values of AST/ALT ratio indicate a lower likelihood of having the metabolic syndrome (*p *= 0.05, and *p *= 0.09, respectively), after adjusting for age, sex, body mass index, physical activity status, and smoking habits of the participants. Ïn the other hand, in people in the highest tertile of the diet score, the previous relationship was not significant (*p *= 0.51). The aforementioned relationships remained similar even when the quantity of alcohol drinking was taken into account.

**Table 3:** Results from logistic regression analyses that evaluated the role of liver enzymes on the presence of the metabolic syndrome, by the level of adherence to the Mediterranean diet.

*Away from the Mediterranean diet (n = 1013)*	Odds ratio	95% confidence interval
AST/ALT ratio (per 0.1 unit)	0.89	0.80–1.00
AST (per 1 IU/L)	0.93	0.88–0.99
ALT (per 1 IU/L)	1.01	0.95–1.07
γ-GT (per 1 IU/L)	0.99	0.95–1.03
*Moderate adherence to the Mediterranean diet (n = 1015)*		
AST/ALT ratio (per 0.1 unit)	0.90	0.78–1.01
AST (per 1 IU/L)	0.96	0.90–1.03
ALT (per 1 IU/L)	0.99	0.92–1.07
γ-GT (per 1 IU/L)	1.02	0.96–1.07
*Close to the Mediterranean diet (n = 1014)*		
AST/ALT ratio (per 0.1 unit)	0.93	0.76–1.13
AST (per 1 IU/L)	0.93	0.76–1.15
ALT (per 1 IU/L)	1.01	0.83–1.22
γ-GT (per 1 IU/L)	1.19	0.99–1.33

All models were also adjusted for age, gender, physical activity status, smoking and body mass index of the participants.

Moreover, no significant associations were observed when AST, ALT and γ-GT were independently tested against the presence of the metabolic syndrome in a stratified by diet pattern analysis.

## Discussion

Studying a community-based representative sample of adult males and females without any clinical evidence of cardiovascular or other chronic disease, we observed that the metabolic syndrome was related with a lower AST/ALT ratio. However, the most interesting finding is that the latter relationship was altered when people were stratified by the level of adherence to the traditional Mediterranean diet. Particularly, AST/ALT ratio constitutes a marker of the metabolic syndrome only among people that report moderate to low adherence to the Mediterranean diet, irrespectively of the quantities of alcoholic beverages drinking and to the best of our knowledge, this is the first study to point out such a thing. Although it is known that a high AST/ALT ratio may be indicative of severe liver damage from alcohol, it is not the case in our study as the ratio of aminotransferases is not a combined with elevated liver enzymes but the mean aminotransferase levels both in people with or without metabolic syndrome were around 25 IU/L [13]. Moreover, only AST/ALT ratio and not AST, ALT or γ-GT, was a marker of the syndrome when dietary habits of people were taken into account, denoting that aminotransferases concentrations should be considered together when evaluating the development of the metabolic syndrome. Additionally, in our study, both men and women in the higher quartile of the AST/ALT ratio, where ALT is considerably lower than AST (or AST considerably higher than ALT), have less likelihood to have the metabolic syndrome. Why AST behaves differently than ALT so the ratio becomes important is not clarified; nevertheless, there are some physiological differences that may explain it. ALT has low concentrations in skeletal muscle and kidneys, so it is more specific for liver damage than AST that is diffusely represented in the heart, skeletal muscle, kidneys, brain and red blood cells. ALT and AST both require pyridoxal-5'-phosphate (vitamin B_6_) in order to carry out the reaction of transfer of α-amino groups from aspartate and alanine to the α-keto group of ketoglutaric acid but pyridoxal-5'-phosphate affects more ALT. Finally, from observational studies ALT seems to correlate more with insulin resistance but the nature of this connection need to be elucidated [28,29]. The associations of aminotransferases levels with the metabolic syndrome or its constituents have already been reported [30,31]. Particularly, insulin resistance study show that liver enzymes may predict metabolic syndrome and additionally, that not only ALT but aminotransferase ratio also may be used as a marker for metabolic syndrome [11]. Liver enzymes and the related pathology of NAFLD are associated with metabolic syndrome through many metabolic disorders like overweight and obesity, dyslipidemia, diabetes and hypertension, while insulin resistance is now considered the main link between metabolic disturbances and elevated liver enzymes [30-33]. Nevertheless, our study has revealed an association of metabolic syndrome only with AST/ALT ratio and not also with specific liver enzymes, as the abovementioned studies. Regarding γGT, although it was not correlated with metabolic syndrome as a biniary entity, a trend analysis has shown that it is related with the number of metabolic components and this is in agreement with previous studies [8,31]. The majority of preceding related studies found no relationship between AST and metabolic disorders. However, most of these studies have positively correlated ALT with metabolic syndrome; on the other side, in our study, men had considerably low levels of ALT (below the cut-off of 30 IU/L for men) in both groups -those with and those without metabolic syndrome [34,35]. In women, the insignificant difference of ALT levels between those with and without the metabolic syndrome may be partially explained by the vast distribution of ALT. Moreover, because of the good level of adherence to the Mediterranean diet of the studied women and the protective effect of this diet to various metabolic abnormalities (i.e., hypertension, dyslipidemia, diabetes), one can speculate that the burden of the metabolic syndrome in our female sample has a more "benign" profile compared with other populations.

Indeed, the favourable effect of Mediterranean diet to the metabolic syndrome has been demonstrated by a number of studies, including ATTICA [15,16,36]. However, it is not lucid if Mediterranean diet could have as well, a beneficial role to -metabolic syndrome related- liver fat accumulation and relative biomarkers. It is well-known that by managing obesity, hypertriglyceridemia, diabetes mellitus and by moderating alcohol consumption, liver fat may be prevented or minimized. Interventions like drugs, diet and exercise, that reduce insulin resistance, may decrease the amount of fat in the liver and normalize aminotransferase levels [37-39]. As Mediterranean diet is related with less insulin resistance, one can assume that such a diet could improve liver fat and enzymes [40]. Our study has established a relationship between Mediterranean diet and AST/ALT ratio, apart from alcohol consumption and other potential confounders but further enquiry will show if insulin resistance is the basic mediator of this relation. The reason why AST elevation occurs independently than ALT elevation when someone has a great adherence to the Mediterranean diet is not clear but maybe it is due to the abovementioned differences of aminotransferase functions [28].

Furthermore, additional investigation is needed in order to comprehend how Mediterranean diet affects the relation of aminotransferase ratio with metabolic syndrome. A number of underlying mechanisms may interpret the phenomenon of the dissociation of the abovementioned parameters. It is already mentioned that ALT and AST/ALT are markers closely related to pathology of NAFLD, meaning with liver fat storage [9]. Adherence to Mediterranean diet may well be capable of protecting from building up more liver fat, even in people with metabolic syndrome or when the probability of having the metabolic syndrome increases. It is possible, that this type of diet reduces the effect on the liver, of cytokines and adipokines secreted from the extra adipose tissue. These mediators could affect the transcriptional factors peroxisome proliferator-activated receptors (PPARs) and the transcription factor sterol regulatory element binding protein-1c (SREBP-1c) of hepatocytes, meaning the regulators of mitochondrial fatty oxidation and liver fat synthesis [41]. Furthermore, ALT and AST/ALT are markers of insulin resistance and "ATTICA" and other studies have already shown that Mediterranean diet is associated with lower levels of insulin resistance and this can be true also in people with metabolic syndrome [36,40]. Moreover, hypoadiponectinemia which characterizes metabolic syndrome and its components and seems to correlate also with NAFLD, may be altered by a Mediterranean diet. In fact, there are indications that levels of adiponectin are related with this type of diet. [42]

On the other hand, Mediterranean diet could minimize the contribution of hepatic inflammation secondary to liver steatosis, to the low-grade inflammation associated with the metabolic syndrome, by reducing, for example, the hepatic production of tumor necrosis factor-α, which triggers the production of other cytokines [43]. By turning down the inflammatory and oxidative processes, Mediterranean diet may also lessen the -derived from liver lipid storage- hepatic insulin resistance and the raise of endogenous glucose production, which in turn may accompany metabolic syndrome. Moreover, specific insulin pathway signalling events are altered in the adipose tissue of patients with NASH compared with non-progressive forms of NAFLD [44]. So, in case of a great adherence to Mediterranean diet, other factors apart from liver fat storage must be recognized as responsible for metabolic syndrome.

The possible advantageous quality of Mediterranean diet to AST/ALT ratio and NAFLD should be further investigated by intervention studies. Additionally, the underlying mechanisms by which this type of diet modifies the relationship of aminotransferases ratio with metabolic syndrome need to be elucidated.

### Limitations

This is a cross-sectional study, so it could not establish causal relationships, but only states hypotheses about the link between liver enzymes and aminotransferase ratio with metabolic syndrome and Mediterranean diet. In discussion, elevated liver enzymes and low AST/ALT ratio were utilized as an estimate of liver fat accumulation, assuming that most cases are markers for NAFLD. By excluding people with HBsAg+ or anti-HCV, this estimation is even more accurate and this is another limitation of our study, that participants did not underwent these measures. Although NAFLD is ideally diagnosed by liver biopsy, histological and ultrasonographic studies of patients referred for unexplained aminotransferase elevations indicate that fatty infiltration of the liver is the cause in 90% of cases and on top, the use of liver enzymes as NAFLD markers is a common methodology in epidemiological cross – sectional surveys, and therefore, our results are comparable. [5,11] Although AST/ALT ratio is inversely linked with liver fat, in a small percentage of asymptomatic subjects, it is also correlated with more severe liver damage and fibrosis, so, the true cause of liver enzyme elevations and the significance of aminotransferase ratio, in the study participants cannot be determined with certainty. Nevertheless, in our study the mean AST/ALT ratio was <2 for people with but also without metabolic syndrome, while severe liver damage (from alcohol) is related with values >2 [13].

## Conclusion

Adherence to the Mediterranean diet is positively correlated with AST/ALT ratio and should be further examined for its favorable impact to non alcoholic fat liver disease. Furthermore, this type of diet modifies the relationship of the liver enzymes ratio with metabolic syndrome; hence in people with a great adherence in Mediterranean diet, alterations in liver enzymes do not affect the likelihood of having metabolic syndrome and vice versa. Our findings pose the need for further investigation on whether Mediterranean diet may have a positive effect in liver biochemistry and liver fat accumulation by moderating metabolic syndrome related mechanisms or its beneficial role to metabolic syndrome could partially be explained by its consequences to liver fat and associated biomarkers.

## Competing interests

The authors declare that they have no competing interests.

## Authors' contributions

NT wrote the paper, DP, CP, CC, designed the study and supervised the data collection, YS, EP, CS critically reviewed the paper. All authors read and approved the final manuscript.
